# Restoring ankle dorsiflexion range of motion in athletes: an individualized clinical decision-making system

**DOI:** 10.3389/fspor.2025.1677383

**Published:** 2025-10-22

**Authors:** Romain Tourillon, Massamba M'Baye, Michelle Smith

**Affiliations:** ^1^ Physiotherapy Department and Motion Analysis Lab, Hôpital de la Tour, Meyrin, Switzerland; ^2^ University Savoie Mont-Blanc, Interuniversity Laboratory of Human Movement Sciences, Universite Jean Monnet Saint-Etienne, Saint-Étienne, France; ^3^ Kin’Aixpert, Viviers du Lac, France; ^4^ Simplexity Performance Solutions, La Motte Servolex, France; ^5^ School of Health and Rehabilitation Sciences, Physiotherapy, The University of Queensland, Brisbane, QLD, Australia

**Keywords:** ankle, range of motion, clinical reasoning, manual therapy, muscle stretching exercises

## Abstract

**Background:**

Ankle dorsiflexion range of motion plays a pivotal biomechanical role within the lower limb with implications both in rehabilitation, injury risk reduction and athletic performance. However, clinicians often lack practical guidance on diagnosing and differentiating the various joints or structures that have been shown to have a role in ankle dorsiflexion range of motion restriction.

**Clinical question:**

To move beyond the “one size fits all approach” paradigm in musculoskeletal rehabilitation, we propose addressing the 2 following questions: (1) How can clinician utilize the weight-bearing lunge test findings to develop a clinical-decision making system for ankle dorsiflexion range of motion assessment? and (2) How can this system guide individualized interventions to restore ankle dorsiflexion range of motion specific to each athlete's needs?.

**Solutions:**

We outline a 3-step framework for improving ankle dorsiflexion range of motion restriction: (1) having a quantitative and qualitative assessment using the weight-bearing lunge test to identify joint or structure involvement, (2) having confirmatory diagnostic testing to pinpoint mobility restrictions of the joint or structures involved, and (3) proposing targeted interventions based on individual findings, ensuring a personalized rehabilitation approach rather than a generalized global protocol.

**Clinical application:**

This rehabilitation practice commentary addresses a notable gap in the existing literature on clinical choices regarding ankle dorsiflexion restriction treatment. By integrating this individual clinical decision-making system, clinicians can enhance rehabilitation and performance optimization beyond standard treatment methods.

## Highlights

### Findings

Ankle dorsiflexion range of motion plays a pivotal biomechanical role within the lower limb with implications for both rehabilitation and athletic performance. However, clinicians often lack practical guidance on diagnosing and differentiating the various joints or structures that can limit dorsiflexion range of motion.

### Implications

Ankle dorsiflexion restriction can be effectively managed by using an individualized clinical decision-making approach consisting of a 3-step framework with the weight-bearing lunge test as its foundation. It involves quantitative and qualitative assessment to identify joint or structure involvement, confirmatory diagnostic testing to pinpoint mobility restrictions, and targeted interventions based on individual findings, ensuring a personalized rehabilitation approach.

### Caution

There are no interventional data demonstrating that this clinical decision-making system with multiple treatment possibilities results in superior ankle dorsiflexion ROM gains than a generalized global protocol.

## 1 Introduction

Ankle dorsiflexion range of motion (ROM) is fundamental for numerous activities of daily living, such as walking, descending stairs (1, 2), and sporting movements (e.g., sprinting, cutting, and squatting) (3–5). During the stance phase of ambulatory tasks, the foot-ankle complex operates around 3 primary axes of rotations, known as the “*heel rocker”, “ankle rocker”* and *“forefoot rocker”* (2, 6)*.* The “*ankle rocker”* describes the transition from foot flat to maximum tibial dorsiflexion until the heel begins to lift (6), representing a critical shift from force absorption to propulsion. The axis of rotation occurs at the talocrural joint, playing a pivotal biomechanical role (2).

Previous biomechanical studies have demonstrated that restricted ankle dorsiflexion ROM after foot-ankle traumatic injuries is related to a disruption in normal talar arthrokinematics, leading to sensorimotor and functional impairments (7, 8). Such impairments can compromise foot-ankle landing mechanics by preventing the foot from reaching its closed-pack position during full loading (9). Additionally, ankle dorsiflexion ROM deficit limits the ability to fully flex the knee during weight-bearing, increasing knee-valgus displacement and peak ground reaction forces (GRF) during landing, squatting and step down (3, 10–13). This suggests that restricted dorsiflexion may affect force absorption capacity, potentially increasing ankle and knee musculoskeletal loading due to sagittal and/or frontal-plane compensations. Consequently, ankle dorsiflexion deficit is a risk factor for various lower-limb injuries including lateral ankle sprain and chronic ankle instability (14–16), Achilles tendinopathy (17, 18), metatarsal bone stress fracture (19), plantar heel pain (20), and patellar tendinopathy (21, 22).

Beyond rehabilitation, ankle dorsiflexion ROM could also impact athletic performance as the tibia functions as an organic protractor guiding force applications against the ground (4). For example, athletes with greater dorsiflexion angles (i.e., triple flexion) demonstrate superior deceleration capacity during high-intensity cutting maneuvers, enabling them to dynamically lower their center of mass position when braking (23). Additionally, the “*ankle rocker”* ROM and stability can modulate braking GRF magnitude during deceleration (23, 24) while influencing the ratio of forces during acceleration (4, 25). Given its importance for both injury management and biomechanical efficiency for performance optimization, restoring ankle dorsiflexion ROM in athletes is essential.

Despite its clinical relevance and recognition as the gold-standard for dorsiflexion ROM assessment (16, 26), many clinicians do not utilize the weight-bearing lunge test (WBLT) in their practice (27, 28). The absence of quantitative measurement and qualitative information the WBLT can offer often results in a generalized “one fits all approach” treatment, incorporating generic and global interventions, such as stretching exercises, manual therapy and massage for every athlete (28, 29). A previous randomized controlled trial employing a pragmatic clinical methodology—rather than a one-size-fits-all research protocol—adapted manual therapy techniques to individual treatment responses and demonstrated a large effect size in improving dorsiflexion ROM (30). This supports the need for researchers and clinicians to adopt a systematic and individualized approach to address the specific anatomical structure(s) [e.g., non-contractile (31–34), contractile (35–37) or neural (38–40) tissues] that are restricting ankle dorsiflexion ROM in athletes (see Supplementary Figure S1).

## 2 Clinical questions

To move beyond the “one size fits all approach” paradigm in musculoskeletal rehabilitation, we propose addressing the following two questions: (1) How can clinician utilize the WBLT as a clinical-decision making system for ankle dorsiflexion ROM assessment? and (2) How can this system guide individualized interventions to restore ankle dorsiflexion ROM specific to each athlete's needs?

Ankle dorsiflexion restriction does not stem from a single cause but rather from multiple contributors that necessitate distinct therapeutic approaches (see Supplementary Figure S1). However, clinicians often lack practical guidance on diagnosing and differentiating these restrictions based on their patients' clinical presentations. Therefore, we aim to provide such guidance in our rehabilitation practice commentary based on existing research and our own experience evaluating and improving ankle dorsiflexion ROM in various musculoskeletal and sports injuries. Our clinical decision-making system presents a structured 3-step framework, utilizing the WBLT as the cornerstone of clinical reasoning. This framework includes: (1) quantitative and qualitative evaluation using the WBLT to identify potential joint and structure involvement in the dorsiflexion ROM restriction; (2) confirmatory diagnostic testing to pinpoint specific mobility restrictions within contractile, non-contractile and neural tissues; and (3) selection of targeted interventions based on individual assessment findings, for a tailored rehabilitation approach. This framework should be used in the acute or chronic phase of rehabilitation of any athlete that suffers from a dorsiflexion ROM deficit following a foot-ankle injury.

## 3 Clinical decision making-system

### 3.1 Step 1: conducting a quantitative and qualitative assessment of ankle dorsiflexion ROM

The initial assessment of ankle dorsiflexion ROM should include the WBLT (41). Although widely accepted in clinical practice, various versions and variations of the WBLT have emerged (26). We propose four standardized rules to enhance reliability and validity: (1) ensure weight-bearing on the tested leg during a tandem stance position (26); (2) standardize the position of the back foot with the heel raised off the floor to minimizes the influence of triceps surae or joint restrictions in the non-tested (back) leg (42); (3) avoid any lower limb movements compensations such as medial hip rotation and knee valgus that may influence ankle dorsiflexion ROM by aligning the patellar (when lunging forward) with an extension of this line up the wall (43); (4) palpate the posterior heel/flat pad during dorsiflexion to carefully monitoring heel lift off the ground and stop the test when the first movement is felt/observed. Following these principles ensures a reliable quantification of ankle dorsiflexion ROM, either through the toe-to-wall distance (44) or tibial inclination degrees (45). Using previous published MDC, clinically relevant impairments are defined as asymmetries exceeding 1.5 cm toe-to-wall distance or 4.7° tibial inclination angle (26, 44, 45). Our clinical experience suggests normative values for ankle dorsiflexion ROM of >9–10 cm and >40–42°.

**Figure 1:**
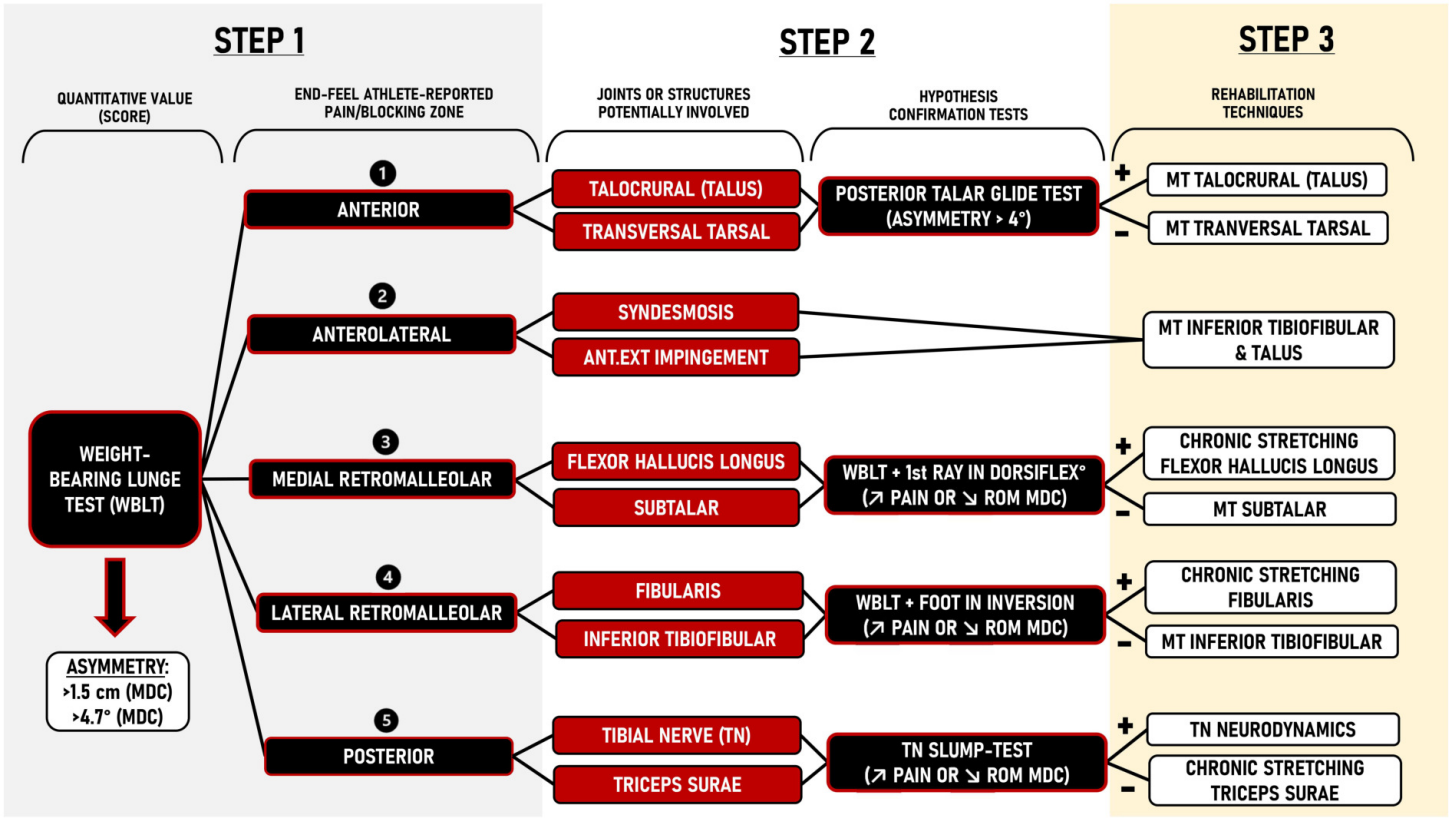
Ankle dorsiflexion range of motion clinical decision-making system using a 3-step framework. MDC, minimal detectable change; ANT.EXT, antero-external; ROM, range of motion; SLR, straight leg raise; MT, manual therapy; TN, tibial nerve; DORSIFLEX°, dorsiflexion.

Beyond the quantitative value (distance or angle measure), clinicians should assess patient-reported symptoms (qualitative aspects) during the WBLT, such as areas/zones of pain/discomfort or a blocking sensation, as these influence clinical decision-making (Figure 1). Based on literature and clinical experience, common pain or blocking sensation zones during the WBLT include:

• Anterior zone: talocrural joint [e.g., posterior talar glide (31, 32)] or transversal tarsal joint motion restriction (34).
• Anterolateral zone: inferior tibiofibular joint motion restriction (33).
• Medial retromalleolar zone: flexor hallucis longus (FHL) tendon tightness (37) or subtalar joint motion restriction (34).
• Lateral retromalleolar zone: potential fibularis tendon tightness (35) or inferior tibiofibular joint motion restriction (33).
• Posterior zone: triceps surae tendon tightness (36) or tibial nerve mechanosensitivity (38–40).

It is important to mention that, while less common, bony osteophytes can also lead to anterior or anterolateral pain/blocking during the WBLT. This cause of pain and/or restriction can be identified by a hard end-feel during passive dorsiflexion ROM that can be confirmed on radiographs and may require surgical intervention (46).

### 3.2 Step 2: confirming the joints/structures involved in ankle dorsiflexion ROM restriction

The hypotheses regarding joint or structural involvement should be systematically tested using specific confirmation tests (Figure 1 & see Supplementary Figure S2). It is important to acknowledge that athletes may experience multiple zones of pain or blocking sensation (e.g., anterior and medial retromalleolar) or can change zones during the treatment, but the clinical reasoning process remains consistent.

#### 3.2.1 Anterior pain/blocking

Anterior restriction is the most common limitation, suggesting involvement of the talocrural (31, 32), or transversal tarsal joints (34). Given that a posterior talar glide at the talocrural joint is an accessory motion essential for dorsiflexion, and a radiographic study demonstrated that 90% of dorsiflexion ROM occurs at the talocrural joint (34), its restriction warrants clinical investigation (31, 32). The posterior talar glide test (PTGT) is a highly reliable diagnostic tool (ICC = 0.94) for assessing posterior gliding of the talus (31, 32). This test is performed with the athlete seated on a table and an electronic inclinometer (e.g., smartphone) fixed on the tibia. The foot is in maximal dorsiflexion while the examiner stabilizes the talus and passively flexes the knee until a firm end-feel is encountered (31). The angle of passive knee flexion provides an indirect estimate of posterior talar glide (see Supplementary Figure S2). Asymmetries up to 4.7° indicate clinically relevant impairments and suggest the need for specific manual therapy treatment (see step 3) (Figure 1). A posterior talar glide restriction can also be confirmed by performing an antero-posterior talar mobilization assessment. If the PTGT or antero-posterior talar mobilization assessment is similar between sides, alternative restrictions to dorsiflexion ROM must be considered. Specifically, anterior blocking may also stem from limited motion in the transversal tarsal (navicular and cuboid) joints. A useful clinical tip involves applying a downward glide to the navicular and cuboid during the WBLT to determine whether this maneuver alleviates the anterior blocking sensation (see step 3).

#### 3.2.2 Anterolateral pain/blocking

Anterolateral restriction suggests a potential syndesmosis or anterolateral impingement and requires integration of the patient's injury history, symptomatology, and imaging findings to distinguish between these two etiologies. In this case, it is relevant to focus on the restriction of amplitude of the inferior tibiofibular joint to improve ankle dorsiflexion ROM (Figure 1). A cadaver study has shown that a posterosuperior glide to the fibula at the inferior tibiofibular joint improves dorsiflexion ROM (33). A restriction at the inferior tibiofibular joint can assessed by performing a manual mobilization assessment (anterior or posterior glides) of this joint (see step 3). Applying a posterior glide to the fibula during an ankle dorsiflexion and evaluating its effect on ROM and/or symptoms (specifically looking for increased ROM or decreased symptoms) can also indicate if this treatment should be used (Table 1).

**Table 1:** Detailed rehabilitation techniques for improving ankle dorsiflexion range of motion restriction.

Joints/structures involved		Techniques	Volume	Frequency	Video
TALUS	ANT.	**Antero-posterior gliding mobilization** - 1-second rythmic oscillation - Mid-to end ROM (grade III to IV)	**Acute stiffness** 60 gliding/session (4 sets of 15 glides) **Chronic stiffness** 120 gliding/session (8 sets 15 glides)	2 to 3 sessions/week (until WBLT value score is reached)	1
		**MWM (Mulligan) + A-P gliding** - Slow patient dorsiflexion movement - Until first onset of pain or end-ROM with 5 s of gliding maintenance	**Acute stiffness** 40 gliding/session (4 sets of 10 glides) **Chronic stiffness** 60 gliding/session (4 sets 15 glides)		2
TRANSVERSAL TARSAL	ANT.	**Caudal gliding mobilization** - 1-second rythmic oscillation - Mid-to end ROM (grade III to IV)	**Acute stiffness** 60 gliding/session (4 sets of 15 glides) **Chronic stiffness** 120 gliding/session (8 sets 15 glides)	2 to 3 sessions/week (until WBLT value score is reached)	3
		**MWM (Mulligan) + caudal gliding** - Slow patient dorsiflexion movement - Until first onset of pain or end-ROM with 5 s of gliding maintenance	**Acute stiffness** 40 gliding/session (4 sets of 10 glides) **Chronic stiffness** 60 gliding/session (4 sets 15 glides)		4
INFERIOR TIBIOFIBULAR	ANT LAT.	**Antero-posterior gliding mobilization** - 1-second rythmic oscillation - Mid-to end ROM (grade III to IV)	**Acute stiffness** 60 gliding/session (4 sets of 15 glides) **Chronic stiffness** 120 gliding/session (8 sets 15 glides)	2 to 3 sessions/week (until WBLT value score is reached)	5
		**MWM (Mulligan) + A-P gliding** - Slow patient dorsiflexion movement - Until first onset of pain or end-ROM with 5 s of gliding maintenance	**Acute stiffness** 40 gliding/session (4 sets of 10 glides) **Chronic stiffness** 60 gliding/session (4 sets 15 glides)		6
FLEXOR HALLUCIS LONGUS	MED RM.	**Chronic stretching (MTU)** - WBLT with 1st ray in max. dorsiflexion - End ROM between “point of discomfort to onset of pain"	High-intensity & low rest interval > 200 s of time under stretch/session (e.g., 3 sets of 75 s with 30 s of rest)	5 to 7 sessions/week (>1,200 s of time under stretch/week)	7
SUBTALAR	MED RM.	**Medio-lateral or latero-medial gliding mobilization** - 1-second rythmic oscillation - Mid-to end ROM (grade III to IV)	**Acute stiffness** 60 gliding/session (4 sets of 15 glides) **Chronic stiffness** 120 gliding/session (8 sets 15 glides)	2 to 3 sessions/week (until WBLT value score is reached)	8
					9
INFERIOR TIBIOFIBULAR	LAT RM.	**Postero-anterior gliding mobilization** - 1-second rythmic oscillation - Mid-to end ROM (grade III to IV)	**Acute stiffness** 60 gliding/session (4 sets of 15 glides) **Chronic stiffness** 120 gliding/session (8 sets 15 glides)	2 to 3 sessions/week (until WBLT value score is reached)	10
		**MWM (Mulligan) + P-A gliding** -Slow patient dorsiflexion movement - Until first onset of pain or end-ROM with 5 s of gliding maintenance	**Acute stiffness** 40 gliding/session (4 sets of 10 glides) **Chronic stiffness** 60 gliding/session (4 sets 15 glides)		11
FIBULARIS	LAT RM.	**Chronic stretching (MTU)** - WBLT with foot in inversion position - End ROM between “point of discomfort to onset of pain"	**High-intensity & low rest interval** > 200 s of time under stretch/session (e.g., 3 sets of 75 s with 30 s of rest)	5 to 7 sessions/week (>1,200 s of time under stretch/week) (until the WBLT value score is reached)	12
TRICEPS SURAE	POST.	**Chronic stretching (MTU)** - WBLT with forefoot in dorsiflexion - End ROM between “point of discomfort to onset of pain"	**High-intensity & low rest interval** > 200 s of time under stretch/session (e.g., 3 sets of 75 s with 30 s of rest)		13
TIBIAL NERVE	POST.	**Neurodynamics** - Patient in slump or straight leg raise position - Foot-ankle positioning in maximal dorsiflexion, abduction and eversion	**Tensioning mobilization** 80 to 100 s of time under stretch/session (e.g., 2 sets of 10 rep with 5 s of tension on each rep)	3 sessions/week (until WBLT value score is reached)	14
		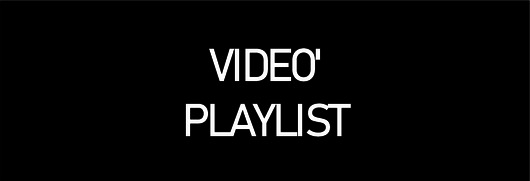	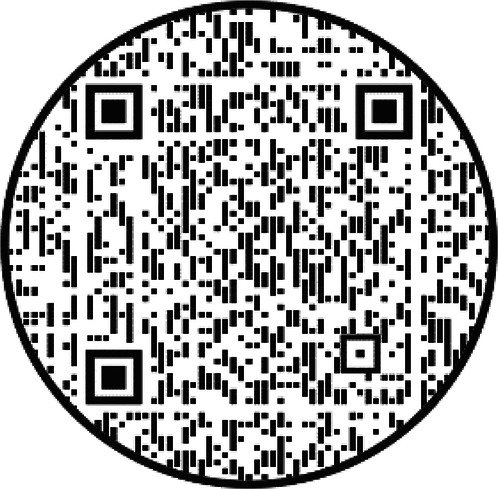		

ROM, range of motion; MWM, mobilization with movement; A-P, antero-posterior; WBLT, weight-bearing lunge test; SEC, seconds; REP, repetitions; ANT, anterior; ANTLAT, anterolateral, P-A, postero-anterior; MTU, muscle-tendon unit; MED RM, medial retromalleolar; LAT RM, lateral retromalleolar; POST, posterior.

#### 3.2.3 Medial retromalleolar pain/blocking

Medial retromalleolar restriction suggests potential involvement of the FHL tendon (37) or the subtalar joint (Figure 1) (34). The FHL originates along the posterior fibula, coursing distally to the muscle-tendon unit (MTU) junction above the fibro-osseous tunnel at the posterior medial ankle (47). Due to the low-lying position of the MTU junction, dorsiflexion of the ankle and the hallux causes distal migration of the tendon and may limit ROM (37). To confirm FHL tendon involvement, we recommend using a modified version of the WBLT by pre-positioning the hallux in maximal dorsiflexion (see Supplementary Figure S2). A ROM reduction (up to 1.5 cm or 4.7°) compared to the initial WBLT indicates FHL tendon tightness, requiring a specific chronic stretching protocol (see step 3) (Figure 1). If this test of FHL involvement is negative, and if medio-lateral or latero-medial subtalar mobilization glides are found to be restricted, improve dorsiflexion range and/or alleviate symptoms (Table 1), a specific focus should be placed on the subtalar joint as this joint also contributes to ankle sagittal plane motion (48).

#### 3.2.4 Lateral retromalleolar pain/blocking

Lateral retromalleolar restriction suggests potential involvement of the fibularis tendon (35) or the inferior tibiofibular joint (see Supplementary Figure S2) (2, 33). The fibularis brevis and longus tendons are both contained within the retro-malleolar groove and may limit ankle dorsiflexion ROM (35, 47). To confirm fibularis tendon involvement, we recommend using a modified version of the WBLT by placing the foot-ankle complex in an inverted position on an inclined plate (∼25°) (see Supplementary Figure S2). A ROM reduction (up to 1.5 cm or 4.7°) compared to the initial WBLT indicates fibularis tendon tightness, requiring a specific chronic stretching protocol (see step 3) (Figure 1). If this confirmation test is negative, a specific focus should be placed on the inferior tibiofibular joint for its impact on ankle dorsiflexion ROM with the same clinical strategies as previously described under anterolateral pain/blocking above (33).

#### 3.2.5 Posterior pain/blocking

Posterior restriction suggests potential triceps surae tightness (36) or tibial nerve mechanosensitivity (38–40). To confirm tibial nerve involvement, we recommend using a modified Straight Leg Raise (SLR) or modified slump-test in which ankle dorsiflexion, rearfoot eversion and forefoot abduction are performed (see Supplementary Figure S2) (49). Asymmetries up to 7.0° (>MDC) (50) or increased posterior leg pain suggest tibial nerve mechanosensitivity, requiring neurodynamic treatment (see step 3) (Figure 1). If the modified Straight Leg Raise or slump-test are negative, focus should be placed on triceps surae tightness, requiring a specific chronic stretching protocol (see step 3) (36).

### 3.3 Step 3: designing an individualized rehabilitation treatment

The final step of this clinical decision-making system involves selecting the appropriate interventions based on individual assessment findings from the confirmatory testing. This ensures that rehabilitation is tailored to the athlete needs (Figure 1 and Supplementary Figure S2).

#### 3.3.1 Talocrural joint mobilizations (posterior glide)

Meta-analyses highlight the efficacy of manual therapy, particularly joint mobilization, in improving ankle dorsiflexion ROM and functional outcomes in individuals with chronic ankle instability (51–54). Among the various manual techniques, antero-posterior talar mobilization (Maitland) and mobilization with movement (MWM – Mulligan) are the most extensively studied and effective techniques (51, 55). Antero-posterior talar mobilizations are performed with the patient in a supine position while applying a posterior glide to the talus using the thumbs or webspace (Table 1). We recommend multiple sets of joint mobilizations using 1-second rhythmic oscillations to end range (Maitland grades III or IV) for approximately 60–120 s (Table 1), with reassessment of ankle dorsiflexion ROM at the end of each set. The frequency and volume of manual therapy play a crucial role in treatment outcomes. Higher doses (e.g., 48 min of manual therapy across 6 sessions vs. 9 min across 3 sessions over two weeks) have been shown to produce significantly greater ROM gains (1, 56–58).

MWM can be performed in non-weight-bearing or weight-bearing positions, with a weight-bearing MWM often considered a progression of an antero-posterior talar mobilizations or non-weight-bearing MWM. A non-weight-bearing MWM is performed in the same position and with the same technique as the antero-posterior talar mobilization. A weight-bearing MWM is performed with the patient standing on a treatment table or in tandem stance with the treatment (front) foot up on a step. A belt (looped around the patient's leg and the therapist), or one of the therapists' hands, is used to apply a postero-anterior force to the distal tibia, while the therapist simultaneously applies an antero-posterior force to talus. The patient performs slow dorsiflexion ROM until the first onset of pain or they reach the end of their ROM, holds this position for a few seconds and then slowly returns to the starting position. The glide is maintained throughout the entire movement (Table 1). Recommended volume per session varies from 40 gliding movements (4 sets of 10 glide) to 60 gliding movements (4 sets of 15 glide) (Table 1), with dorsiflexion ROM reassessed between each set (55). Antero-posterior talar mobilization and/or MWM should be performed 2–3 sessions per week until the desired WBLT score is achieved (providing reassessments identify improvements in dorsiflexion ROM). The manual therapy treatment can be supplemented with a home exercise program (e.g., mimicking the WBLT stopping at end-range or at onset of pain/blocking) to maintain the ROM gains achieved.

#### 3.3.2 Transversal tarsal and subtalar joint mobilizations

Only one study has investigated the effectiveness of transversal tarsal mobilizations on ankle dorsiflexion ROM (59). Transversal tarsal joint mobilizations are performed with the patient in a supine position, stabilizing the rearfoot while applying a caudal/plantar-directed mobilization to the navicular and cuboid (Table 1). This technique can also be done as a weight-bearing MWM as a caudal glide applied to the transverse tarsal joint during dorsiflexion. Subtalar joint mobilizations are conducted in a side-lying position with the patient slightly rolled forward so their foot is slightly angled towards the floor. The talus is stabilized in the tibial mortise in maximal dorsiflexion (or stabilized by the therapist) and the calcaneus is mobilized laterally and medially using the thenar eminence (Table 1). We recommend applying similar manual intensity, volume, frequency, and dosage as used for talar mobilizations.

#### 3.3.3 Inferior tibiofibular joint mobilizations

Research indicates that distal tibiofibular joint mobilizations over multiple sessions improve ankle dorsiflexion ROM (30), whereas a single session yields limited benefits (60, 61). Antero-posterior mobilization is performed in a supine position, stabilizing the distal tibia while applying a posterior glide to the distal fibula with the thenar eminence (Table 1) (60). This technique is recommended for patients with anterolateral pain or blocking sensation during the WBLT (Figure 1). If lateral retromalleolar pain occurs, a postero-anterior mobilization can be performed with the patient in a prone position. The therapist stabilizes the distal tibia and applies an anterior glide to the fibula (Table 1). These techniques can also be performed as MWM in weight-bearing or non-weight-bearing positions (Table 1) (30). We recommend applying a similar manual therapy prescription (intensity, volume, frequency, and dosage) as described previously.

Evidence suggests that a pragmatic, patient-responsive approach could yield superior outcomes compared to standardized uniform protocols. Adapting mobilization techniques to an individual's clinical presentation, as advocated in our model, has demonstrated clinically relevant benefits for ankle dorsiflexion ROM (30). Notably, improvements in dorsiflexion ROM after a single session (+1.7 cm vs. + 1.1–1.2 cm) and across three sessions of individualized manual therapy, were greater than that reported in studies that applied identical manual techniques (high velocity and low amplitude manipulation or MWM) to all participants (30, 62, 63).

#### 3.3.4 Flexor hallucis, fibularis and triceps surae tendon chronic stretching

The effectiveness of stretching in modifying MTU properties and neural adaptations is influenced by three key factors: stretch intensity, total time under stretch, and duration (e.g., weeks) of stretching (64). Meta-analyses support static stretching as an effective strategy to increase ankle dorsiflexion ROM, particularly when restricted by triceps surae tightness (29, 65). We recommend a chronic stretching protocol (duration greater than 2 weeks) (66) for the triceps surae, FHL or fibularis muscle-tendons, adhering to the following principles: (1) stretch intensity should range from “point of discomfort” to “onset of pain”; (2) total time under stretch should reach at least 1,200 seconds per week, using high-intensity, low-rest intervals during stretching sessions (e.g., 3 sets of 75-second stretches with 30-second rest periods, six times per week); (3) the protocol should last a minimum of five weeks until the target WBLT score is achieved. The stretching position should be a weight-bearing lunge with the forefoot in dorsiflexion for the triceps surae, the hallux in maximal dorsiflexion for the FHL, and the foot in inversion for the fibularis (Table 1). If supra-maximal eccentric contractions have shown effects on improving flexibility of the posterior chain (67), it is also possible to consider this modality provided that you have the necessary equipment (heavy-load machines) to be able to target triceps surae, FHL or fibularis muscle-tendons.

#### 3.3.5 Tibial nerve neurodynamics

Only one study has demonstrated that a static stretching protocol targeting the sciatic and tibial nerves—without stretching the triceps surae—effectively increases passive ankle dorsiflexion ROM (40). In cases where posterior pain or blocking is unrelated to triceps surae tightness, we recommend neurodynamic techniques focusing on tibial nerve mobilization, using tensioning exercises in a slump or straight leg raise position (68, 69). Given the variability in reported protocols (68, 70), we suggest a total time under stretch of the nerve of approximately 80–100 s per session by completing 2 sets of 10 repetitions (with 5 s of stretch in each repetition) three times per week (Table 1) (71).

## 4 Conclusion

This commentary addresses a notable gap regarding the lack of guidance in treating ankle dorsiflexion ROM restriction in athletes. Ankle dorsiflexion ROM is vital for rehabilitation and performance, with limited ROM leading to altered biomechanics and injury risk. The WBLT, the gold standard for assessing dorsiflexion ROM, is underutilized. We propose a new clinical decision-making framework involving three steps: quantitatively and qualitatively assessing dorsiflexion ROM, identifying the restriction source (joints, muscle-tendons, or neural tissue), and applying targeted interventions such as joint mobilizations, chronic stretching, and neurodynamic techniques. This individualized and analytical approach could then be followed by functional therapeutic exercises (e.g., single-leg squat, lateral step-down) that aimed to developed lower limb motor control and stability in ankle dorsiflexion position.

## Data Availability

The original contributions presented in the study are included in the article/Supplementary Material, further inquiries can be directed to the corresponding author.

## Supplementary material

Supplementary Figure S1 Anatomical joints and structures within the foot-ankle complex that have been shown to have a role in ankle dorsiflexion range of motion restriction (31–40).

Supplementary Figure S2 Hypothesis confirmation tests of specific anatomical structures that have a role in ankle dorsiflexion range of motion restriction. Abbreviation: MDC, minimal detectable change; ROM, range of motion; WBLT, weight-bearing lunge test; TN, tibial nerve; SLR, straight leg raise; LAT, lateral; MED, medial.
